# The RhoA GTPase regulates Type I Interferon Signaling in Systemic lupus erythematosus

**DOI:** 10.21203/rs.3.rs-3320841/v1

**Published:** 2023-09-20

**Authors:** Wei Fan, Bo Wei, Xuyan Chen, Yi Zhang, Pingping Xiao, Kaiyan Li, Yi qin Zhang, Jinmei Huang, Lin Leng, Richard Bucala

**Affiliations:** the Second Affiliated Hospital of Xiamen Medical College, Xiamen Medical College; Zhongshan Hospital of Xiamen University, Medical College of Xiamen University, Xiamen University; the Second Affiliated Hospital of Xiamen Medical College, Xiamen Medical College; the Second Affiliated Hospital of Xiamen Medical College, Xiamen Medical College; the Second Affiliated Hospital of Xiamen Medical College, Xiamen Medical College; the Second Affiliated Hospital of Xiamen Medical College, Xiamen Medical College; the Second Affiliated Hospital of Xiamen Medical College, Xiamen Medical College; the Second Affiliated Hospital of Xiamen Medical College, Xiamen Medical College; Yale University School of Medicine; Yale University School of Medicine

**Keywords:** Systemic lupus erythematosus, RhoA, type I IFN, RhoA/ROCK inhibitor, Pathologensis

## Abstract

**Objective.:**

Systemic lupus erythematosus (SLE) is an autoimmune disorder characterized by abnormal activation of the type I interferon (IFN) pathway, which results in tissue inflammation and organ damage. We explored the role of the RhoA GTPase in the type I IFN activation pathway to provide a potential basis for targeting GTPase signaling for the treatment of SLE.

**Methods.:**

Total RNA was extracted from peripheral blood mononuclear cells (PBMCs) of SLE patients and healthy controls, and the mRNA expression levels of RhoA and IFN-stimulated genes were measured by SYBR Green quantitative reverse transcriptase-polymerase chain reaction. IFN-stimulated response element (ISRE)-luciferase reporter gene assays and Western blotting were conducted to asssess the biologic function of RhoA. An Enzyme-Linked Immunoassay (ELISA) measured C-X-C motif chemokine ligand 10(CXCL10)protein expression.

**Results.:**

Our studies demonstrated that the expression of RhoA in the PBMCs of SLE subjects was significantly higher than healthy controls and positively correlated with type I IFN scores and type I IFN-stimulated gene (ISGs) expression levels. SiRNA-mediated knockdown of RhoA and the RhoA/ROCK inhibitor Y27632 reduced the activity of the type I IFN-induced ISRE, the signal transducer and activator of transcription 1 (STAT-1) phosphorylation, and the expression of CXCL10 and 2’-5’-oligoadenylate synthetase 1(OAS1). Finally,we verified that Y27632 could significantly down-regulate the OAS1 and CXCL10 expression levels in PBMCs of SLE patients.

**Conclusion.:**

Our study shows that RhoA positively regulates the activation of the type I IFN response pathway. Reducing the expression level of RhoA inhibits the abnormal activation of the type I IFN system, and the RhoA/ROCK inhibitor Y27632 decreases aberrant type I IFN signaling in SLE PBMCs, suggesting the possibility of targeting the RhoA GTPase for the treatment of SLE.

## Background

Systemic lupus erythematosus (SLE) is a multisystem autoimmune disease that causes immune dysregulation and chronic inflammation, leading to progressive end-organ damage (1). Studies over several decades have underscored the pathogenic complexity of SLE, but type I IFN produced by innate immune cells and activating the adaptive immune system are considered important for the initiation and maintenance of the disease (2, 3). Sustained activation of the type I IFN pathway leads to excessive production of numerous, tissue damaging inflammatory cytokines, which mediate many of the long term pathologic sequelae in skin, kidneys, and other organ systems (4–7).

The majority of patients with SLE exhibit abnormal expression of multiple type I IFN-inducible genes, known as the type I IFN signatures (2, 3, 8). The IFN expression signature determined in the tissues or serum of lupus patients are associated with pathogenesis, clinical manifestations, and disease activity (9–12). Previous reports have shown that therapeutic use IFN-a, a major type I IFN family member, can induce an SLE-like syndrome, while blocking the action of type I IFNs or their common receptor prevents immunological dysfunction and reduces tissue damage in SLE (13–16).

RhoA is a member of the Rho-GTPases family that has a crucial role in a variety of biologic processes, including cell adhesion and migration, apoptosis, and in the regulation of immunologic functions (17–20). RhoA exerts its major functions by activating Rho-associated serine/threonine protein kinases (ROCKs), which include the two isoforms: ROCK1 and ROCK(19).Activation of the RhoA/ROCK pathway in autoimmune disease has been well described, although its specific contribution to SLE is only beginning to emerge (17). Previous studies have reported that inhibition of RhoA/ROCK signaling significantly reduced anti-dsDNA antibody levels in lupus-prone NZB/NZW F1 mice, effectively alleviated renal damage and reducing mortality (21). In our project,*RhoA* mRNA expression further has been shown to be higher in the PBMCs of lupus patients when compared to healthy controls, and expression levels correlate positively with type I IFN scores and interferon-induced genes, including CXCL10, OAS1, IFIT3 and MX1. The PBMCs of a majority of lupus patients also exhibit up-regulated ROCK activity(22).

We hypothesize that the overexpression of RhoA leads to pathogenic activation of type I IFN pathway, with consequences for immune activation in SLE. Accordingly, we examined the activation state of RhoA and its downstream actions in the PBMCs of lupus patients, and assessed the consequences of RhoA pathway inhibition on selected type I IFN-induced genes relevant to immune dysregulation.

## Materials and Methods

### Study subjects.

We enrolled 36 SLE patients admitted to the Second Affiliated Hospital of Xiamen Medical college according to the American College of Rheumatology 1997 revised criteria for SLE. Thirty age- and sex-matched healthy controls were drawn from healthy volunteers with no personal history of autoimmune disease or immunosuppressive therapy. All participants signed written informed consent for this study. The study was performed according to the current National Health and Family Planning Commission of China ethical standards and approved by the hospital ethics committee.

### Study samples.

Approximately 15–25 ml of venous blood was drawn from all study subjects into heparinized tubes and centrifuged at 2000 rpm for 10 min at 4°C to extract plasma, which was stored at −80°C. PBMCs were isolated from heparinized blood by density-gradient centrifugation using Ficoll-Paque Plus medium (GE Healthcare) in accordance with the manufacture’s instructions.

### Cells and reagents.

Human THP1 monocytes or PBMCs were cultured in RPMI 1640 medium (Gibco) supplemented with 10% fetal bovine serum (FBS;Gibco) and 1% penicillin-streptomycin (Hyclone,USA in a humidified atmosphere containing 5% CO_2_ at 37°C. The pCMV6-RhoA plasmid overexpressing human-*RhoA* and pCMV6-Ctrl plasmid were purchased from ORiGENE. The two plasmids were confirmed by DNA sequencing and were prepared for transfection using an EndoFree Plasmid Maxi kit (Qiagen). The correct mRNA and protein expression of the clone plasmid were confirmed by reverse transcription-quantitative PCR (RT-qPCR) and Western blot analysis, respectively. RhoA small-interfering RNA (siRNA) sequences and siRNA Universal negative control were obtained from Sigma-Aldrich. The siRhoA sequences were: sense: 5′-CAGAUACCGAUGUUAUACU-3′ and antisense: 5′-AGUAUAACAUCGGUAUCUG-3′.

### Cell Transfection and stimulation.

HEK-293 and THP1 cells were transfected with siRNAs (200 nM) or plasmid (4 μg/mL) and their controls using Lipofectamine RNAiMAX or Lipofectamine 2000 reagent following the manufacturer’s recommendations (Invitrogen). At 24–48 hours after transfection,the cells were stimulated with IFN-a (1,000 units/mL or 1,000U/mL, PBL Interferon Source) for 6 hours. The RhoA/ROCK inhibitor Y27632 (30,60,90 μM, Beyotime) was added 45 minutes before stimulation with IFN-a (1,000 units/mL,PBL Interferon Source).

### Dual-luciferase reporter gene assay.

HEK-293T cells were cultured in a 96-well plate and co-transfected with siRhoA (200 nM) or RhoA expression plasmids (4 μg/mL), or their controls together with a mixture of Renilla vector (10 ng, Promega) and ISRE-luciferase reporter gene vector (100 ng, Clontech) for 24 hours. IFN-a(1,000 units/mL) then was added for an additional 6 hours of incubation. The cells were harvested and lysed, and luciferase activity was measured using the Dual-Luciferase Reporter Assay System (Promega) and CYTATION3 (BioTek) instrumentation. The ratio of firefly luciferase activity to Renilla luciferase activity was calculated for each well. All experiments were performed in triplicate.

### RNA extraction and real-time PCR (RT–PCR).

Total RNA was isolated using the RNeasy Mini kit (Qiagen). To quantify mRNA expression, complementary DNA (cDNA) was synthesized from 500 ng of total RNA with the SuperScriptIII RT Reagent kit (Invitrogen) and amplified by real-time PCR (iTaqTM Universal SYBR Greensupermix; Bio-RAD). The endogenous expression of GAPDH was used as the internal control. The relative expression levels were calculated using the 2-ΔΔCt method. All of the experiments were performed on a ViiA7 Real-Time PCR System (Applied Biosystems).

The primers for RT-PCR were as follow: for GAPDH, forward 5’-CTCCTCCTGTTCGACAGTCA-3’and reverse 5’-CAATACGACCAAATCCGTT

G-3’; for RhoA, forward 5’-TCTTCGGAATGATGAGCAC-3’and reverse 5’-CTTTGGTCTTTGCTGAACAC-3’; for IFIT3, forward 5’ –TGAGGTCACCAAGAATTCCCTG-3’ and reverse 5’-CAATCTGGTTACACACTCTATCTTC-3’; for OAS1, forward 5’-GAAGGCAGCTCACGAAAC-3’and reverse5’-TTCTTAAAGCATGGGTAATTC-3’, for MX1, forward 5’-GGGTAGCCACTGGACTGA-3’and reverse 5’-AGGTGGAGCGATTCTGAG-3’.

### Calculation of type I IFN scores.

Type I IFN scores were calculated according to the relative expression (RE) of the type I IFN-inducible genes MX1, OAS1, IFIT3, and CXCL10. The mean ± SD level of these genes in the healthy control (hc) group (mean_hc_ and SD_hc_) were used to standardize the expression levels per gene for each study subject. The type I IFN scores were calculated by summing up the individual RE of each gene after normalization to the healthy control value as follows: ∑(RE_subject_ − Mean_hc_)/SD_hc_ (23, 10). An IFN-score was regarded positive when it was higher than the mean + 2SD of HC values (11).

### Enzyme-linked immunosorbent assay (ELISA).

The CXCL10 concentrations of plasma samples and cell culture supernatants were analyzed by specific ELISAs (R&D Systems) in accordance with the manufacturer’s instructions.

### Western blotting.

HEK-293T cells were seeded into 6-well plates and 5×10^5^ HEK-293T cells per well transfected with siRhoA (200 nM) or RhoA over-expression plasmids (4 μ/mL), or their controls; 48 hours after incubation, the cells were treated with IFN-a (1,000 units/mL) for an additional 6 hours or RhoA/ROCK inhibitor Y27632 (60 μM, Beyotime ) was added for an additional 45 minutes before adding IFN-a. The cells then were harvested and lysed at different time points and subjected to sodium dodecyl sulfate-polyacrylamide gel electrophoresis and transferred onto polyvinylidene difluoride membranes for immunoblotting followed by protein detection with SuperSignal West Femto Maximum Sensitivity Substrate (Pierce). The visualized proteins were scanned and signal intensities quantified using Image J. The specified primary antibodies were directed against RhoA (Santa Cruz Biotechnology, diluted 1:200), and total and phosphorylated STAT1 and STAT2, (Proteintech, diluted 1:3000), and β-actin (Abcam, diluted 1:5000). The secondary antibodies were horseradish-peroxidase (HRP)-linked anti-mouse IgG antibody (Proteintech 1:0000), and HRP-linked anti-rabbit IgG antibody (Proteintech, diluted 1:10000).

### Statistical analysis.

Descriptive data were presented as mean ± standard deviations (SDs). Continuous variables were analyzed using the Student t-test or nonparametric Mann-Whitney test for comparisons of two groups. Correlations were calculated using Spearman r test. Data were analyzed with Prism 5 (GraphPad Software, Inc, San Diego, CA, USA). P values of 0.05 or less were considered statistically significant.

## Results

### PBMC RhoA expression correlates positively with type I IFN scores in SLE patients.

**Figure 1.**
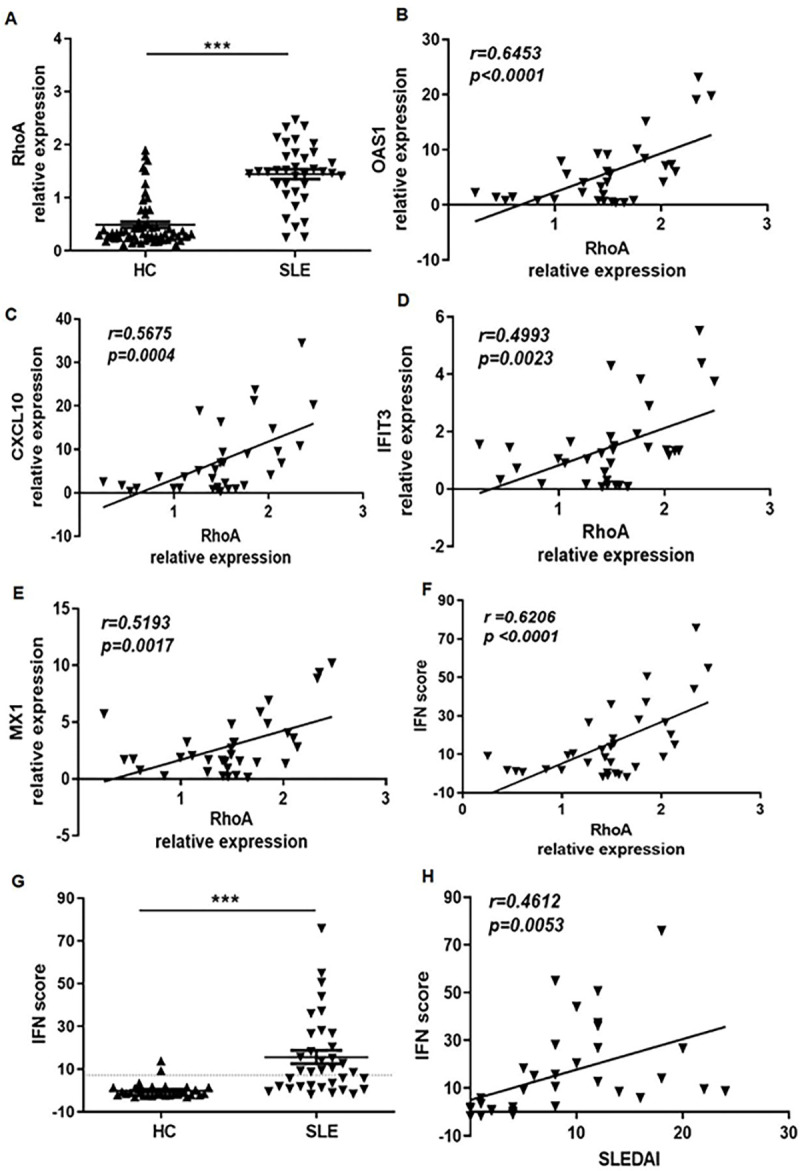
Elevated RhoA expression correlates positively with type I IFN-inducible gene expression and IFN score in SLE patients. (**A**) Significantly higher expression of RhoA mRNA in the PBMCs of SLE patients (n=36) than in healthy controls (HC, n=60). (**B-E**) Correlations between RhoA expression and the type I IFN-inducible genes OAS1, CXCL10, IFIT3 and MX1 in SLE patients. (**F**) IFN scores calculated by integrating the relative expression of the OAS1, CXCL10, IFIT3 and MX1 genes in the SLE and HC groups. (**G**) RhoA was highly expressed in high IFN scores patients. The dotted line represents mean ± 2SD of the HC. (**H**) Positive correlation between RhoA expression levels and SLEDAI values.Bars in A and G show the mean ± SEM. Each symbol represents an individual sample. Expression is defined as relative expression of the gene of interest in comparison to *GAPDH* . *p < 0.05, ***p < 0.001 by Student’s t-test.

We investigated differences in the expression of *RhoA* mRNA in PBMCs between patients with SLE patients and healthy controls by quantitative, real-time PCR (RT-PCR) and observed a 3-fold higher level of *RhoA* mRNA in the lupus group (Fig. 1A). (The characteristics of the two studied cohorts are shown in the Supplementary Table 1). We examined the relationship between RhoA expression in the PBMCs of the SLE group with four well-characterized type I IFN inducible genes: OAS1, CXCL10, IFIT3 and MX1, and observed a significant positive correlation with RhoA expression (Figs. 1B–E). Additionally, RhoA expression correlated with an IFN scores calculated on the basis of the relative expression of these genes (Fig. 1F). As expected, this score was elevated in the SLE versus healthy control group (Fig. 1G)and correlated with the Systemic Lupus Erythematosus Disease Activity Index(SLEDAI) values in our studied population (Fig. 1H).

### RhoA enhances ISRE–luciferase activity and selected type I IFN-induced genes.

**Figure 2.**
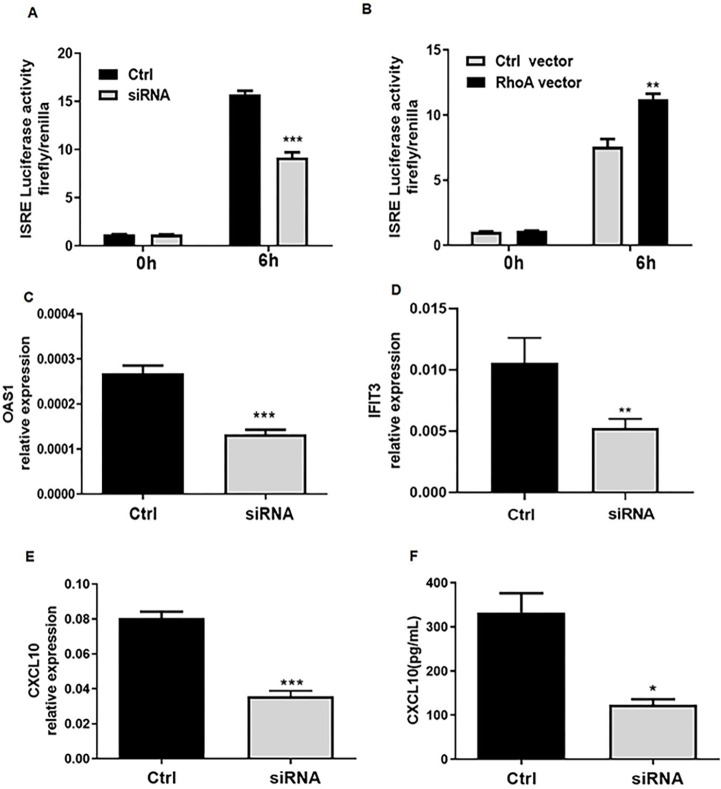
RhoA regulates the type I IFN-stimulated response element (ISRE) and downstream gene expression. (**A**) ISRE activity of HEK293T cells transfected with ISRE-luciferase and a Renilla reporter plasmid together with a negative control (Ctrl) or RhoA targeted siRNA (each at 200 nM). (**B**) ISRE activity of HEK293T cells transfected with RhoA-overexpression or control plasmids (each at 100 ng). At 24 hours after transfection, the were left unstimulated (0 hours) or stimulated with type I IFN (1,000 units/mL, 6 hours). Data in **A** and **B** are expressed as fold-change based on relative luciferase activities (ratio of firefly luciferase to Renilla luciferase). (**C-E**) The relative expression of OAS1, IFIT3 and CXCL10 mRNAs were determined by quantitative PCR. (**F**) The CXCL10 levels in culture supernatants were determined by enzyme-linked immunosorbent assay.Data were assessed in HEK293T(C and D) and THP1 cells(E and F) at 24 hours after transfection of the negative control (Ctrl) or siRNA (200 nM) stimulated with IFN-a(1,000 units/mL) for 6 hours. Bars show the mean ± SEM of 3 independent experiments. *p < 0.05, ***p < 0.001 by Student’s t-test.

We hypothesized that RhoA may upregulate the type I IFN signaling pathway by increasing the activity of interferon response elements (ISRE) in target genes. We quantified ISRE activity in cultured HEK-293T cells transfected with an ISRE-luciferase reporter plasmid and treated simultaneously with siRNA targeting RhoA or a RhoA expression plasmid. We observed that genetic knockdown of RhoA reduced, and forced RhoA expression increased, ISRE–luciferase activity, respectively (Fig. 2A,B). In addition, experimental inhibition or augmentation of RhoA expression had commensurate effects on the expression of OAS1 and IFIT3 (Fig. 2B–D). We found low expression of CXCL10 in HEK-293T cell, so we examined the expression of this chemokine in human in THP-1 monocytes. As expected, siRNA induced RhoA knockdown decreased type I IFN induced CXCL10 mRNA and protein expression levels in THP-1 cells (Fig. 2E, F). Taken together, these findings indicate that RhoA as a positive regulator of ISRE activity and the transcription of the type I IFN-induced genes OAS1, IFIT3, and CXCL10.

### RhoA promotes STAT-1 phosphorylation by type I IFN.

**Figure 3.**
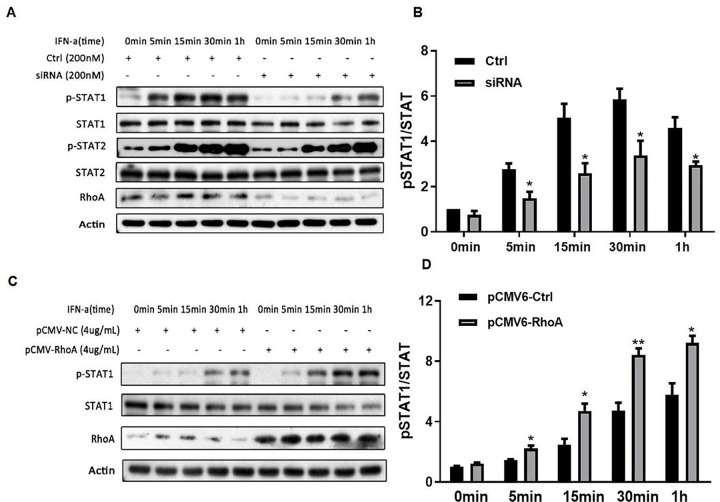
RhoA regulates type I IFN induced STAT-1 phosphorylation. Western blot analysis (A and C) and densitometry histograms(B and D) of cell lysates of cultured HEK293T cells (5×10^5^ cells per well) transfected with siRNA at 200 nM targeting RhoA mRNA (A)or a RhoA expression plasmid vector at 4 ug/mL(C),and their controls(a negative control siRNA or pCMV6-NC) ,and then stimulated with IFN-a(1,000 units/mL) for different times.The staining density histograms are from three independent experiments and values are expressed as the ratios of phosphorylated STAT-1 protein to total STAT-1. The immunoblot of cells transfected with a negative control siRNA (B) or the pCMV6-NC vectors (D) were set at a value of 1. *p < 0.05, **p < 0.01.by Student’s t-test.

The two serine-threonine kinases ROCK1 and ROCK2 are important downstream effectors of Rho GTPases that phosphorylate downstream signaling intermediates. RhoA activation is known to induce the tyrosine phosphorylation of the signal transducer and activator of transcription (STAT) proteins (24, 25). To further clarify the mechanism by which RhoA regulates type I IFN signaling, we tested whether the activating function of RhoA is attributable to the promotion of STAT-1/2 phosphorylation activity. SiRNA genetic knockdown of RhoA reduced and RhoA overexpression increased STAT-1 phosphorylation, respectively, as revealed by Western blotting (Fig. 3A–D). By contrast, the phosphorylation of STAT-2 was not affected by experimental changes in RhoA expression.

### Pharamcologic RhoA/ROCK inhibition reduces type I IFN signaling.

**Figure 4.**
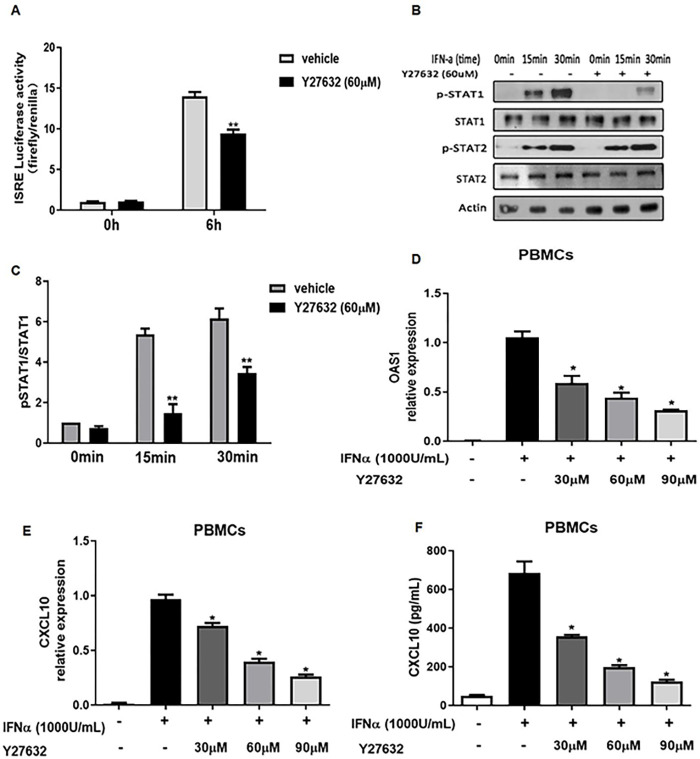
The RhoA/Rock Inhibitor Y27632 downregulates type I IFN signaling. (**A**) HEK293T cells were cotransfected with ISRE-luciferase and Renilla reporter plasmids for 24 hours, then cultured with or without Y27632 (60 μM, 45 min) before the addition of IFα (1,000 U/ml, 6 hrs). Luciferase activity was measured by dual luciferase assay. (**B**) Peripheral blood mononuclear cells (PBMCs) from healthy controls were cultured in the presence or absence of Y27632 (60 μM, 45 min) and stimulated with IFNα (1,000 U/ml) for different times. Western blots show whole-cell lysates harvested at the indicated times. (**C**) Histograms show the ratios of phosphorylated to total STAT-1 at the indicated times. The ratio of pSTAT-1/STAT-1 in the absence of Y27632 and IFNα at 0 minute was set at 1. Bars show the mean±SEM of 3 individual healthy donors; *p < 0.05, **p < 0.01, versus vehicle by Student’s t-test. (**D**) Relative expression of OAS1 and (**E**) CXCL10 mRNA in PBMCs cultured with Y27632 (0–90 μ M, for 45 mins and stimulated with IFNα (1,000 U/mL) for 6 hrs. Results are the relative expression levels of OAS1 and CXCL10 mRNA normalized to endogenous GAPDH mRNA levels. (**F**) CXCL10 levels in PBMC culture supernatants quantified by ELISA. Bars show the mean±SEM of PBMCs values from three different individuals. *p < 0.05 by Student’s t-test for IFNα stimulation in the absence or presence of Y27632.

We next tested if the small molecule RhoA/ROCK inhibitor Y27632 affected the phosphorylation of STAT-1, ISRE–luciferase activity, and the expression levels of ISGs in a manner similar to the knockdown of RhoA. We treated ISRE–luciferase transfected HEK293T cells with Y27632 (60 μM) before stimulation with IFNa(1000U/mL). Both ISRE–luciferase activation and STAT-1 phosphorylation were decreased upon the addition of Y27632 (Fig. 4A–C). We also examined PBMCs treated with Y27632 (60 μM, 45 mins), followed by 6 hours of stimulation with 1,000 units/ml of IFNa. We found that the phosphorylation of STAT-1 was decreased by the prior addition of Y27632 (Fig. 2B,C). Furthermore, there was a dose-dependent decrease in the ability of PBMCs to express OAS1 and CXCL10 mRNA and produce CXCL10 protein (Fig. 2E,F). Taken together, these studies indicate that similar to the genetic knockdown of RhoA, Y27632 decreases the consequences of type I IFN stimulation on RhoA-mediated signaling by reducing ISRE–luciferase activity, STAT-1 phosphorylation, and the expression of the type I IFN induced genes OAS1 and CXCL10.

### RhoA/ROCK inhibition attenuates CXCL10 and OAS1 expression in type IFN score-high SLE PBMCs.

**Figure 5.**
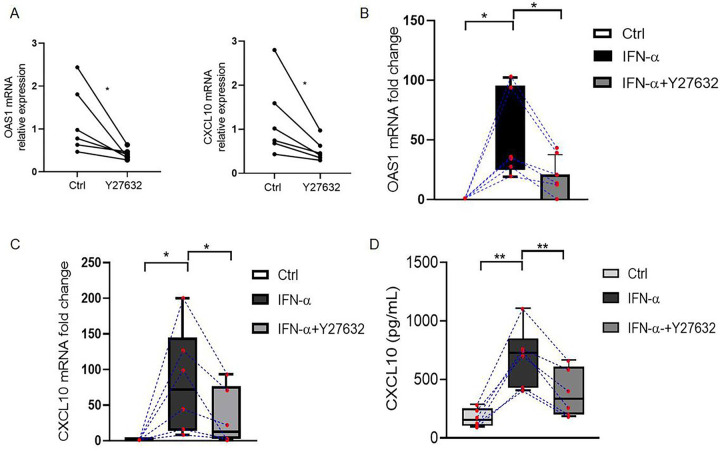
The RhoA/ROCK inhibitor Y27632 reduces type I IFN signaling in lupus PBMCs. (**A**) Relative expression of OAS1 and CXCL10 mRNA in PBMCs obtained from SLE patients (n = 6) and incubated in medium alone or with Y27632 (60 μM, 6 hrs). (**B**) OAS1 and (**C**) CXCL10 mRNA measurements in lysates of lupus PBMCs (n = 6) cultured with or without Y27632 (60 μM, 45 mins) and stimulated with IFNα (1,000 U/mL). Results are the relative expression levels of OAS1 and CXCL10 mRNA normalized to endogenous GAPDH mRNA levels. (**D**) CXCL10 levels in culture supernatants quantified by ELISA. Bars show the mean ± SEM of 6 individual PBMC samples. *p < 0.05 by Student’s t-test.

We next examined if pharmacologic inhibition of RhoA could reduce the type I IFN signaling pathway in PBMCs obtained from lupus patients with high type I IFN scores (see Supplementary, Table 2 for patient characteristics). We incubated PBMCs with Y27632 (60 μM, 6 hrs) and observed a reduction in the expression of OAS1 and CXCL10 mRNA when compared to medium alone (Fig. 5A). The addition of IFNa (1000 U/ml, 6 hrs) to these PBMCs increased baseline OAS1 and CXCL10 mRNA expression, and CXCL10 protein production but these increases were nevertheless significantly reduced by prior treatment with Y27632 (60 μM, 45 mins).

## Discussion

Systemic lupus erythematosus is an autoimmune disease provoked by aberrant and sustained type I IFN responses and elevated levels of pro-inflammatory cytokines, leading to tissue inflammation and critical end-organ damage (26). Despite improvements in treatment during the past several decades, complete control of lupus disease activity is rarely achieved (27). Elevated levels of type I IFN and its gene expression signature correlate with measures of disease activity and clinical flares (28), leading to therapeutic efforts targeting IFN-α, the type I IFN receptor, and downstream signaling molecules (2, 16, 29, 30). Anifrolumab, a monoclonal antibody targeting the type I IFN receptor, has been shown to reduce disease activity in SLE patients (13), although not without risks of excessive immunosuppression and infections (30).

RhoA is a member of the Rho subfamily of GTPases that is rapidly activated by a diverse array of biochemical signals to regulate numerous biological processes, including cytoskeletal reorganization, cell proliferation and differentiation and apoptosis (31–33). The RhoA/ROCK pathway has been implicated in lupus pathology in a prior study that showed that increased phosphorylation of the ezrin, radixin, moesin (ERM) proteins interact with CD44 to promote the adhesion, migration and inflammatory response of T lymphocytes (34). Genetic knockdown of RhoA was observed to suppress the apoptosis of renal tubular epithelial cells in mice with lupus nephritis(35). The RhoA/ROCK inhibitor, Y27632, also attenuates disease development in lupus-prone mice by diminishing T cell production of IL-17 and IL-21 (36), and it reduced serum concentrations of tumor necrosis factor-α (TNF-α), IL-1β and interleukin-6 (IL-6) while increasing levels of of IL-10 (37). Additionally, pharmacologic RhoA/ROCK inhibition reduced the production of anti-dsDNA antibody levels and the responsiveness of B cells to B-cell activating factor receptor (BAFF/ BAFFR) (33, 38),suggesting a role in the differentiation of autantibody producing B lymphocytes in SLE (39).

Previous work has shown that the expression of RhoA is significantly higher in lupus T cells and that targeting RhoA can reduce their production of IL-2 (40). In the present, we examined the influence of the RhoA GTPase in type I IFN signaling and the expression of a subset of type I IFN pathway genes. We confirmed RhoA to be highly expressed in the PBMC population of SLE patients when compared to healthy controls, and especially in those lupus patients with high type I IFN scores. Our study also showed that genetic reduction in RhoA expression or pharmacologic inhibition of RhoA activity reduced activation of the type I IFN pathway, supporting its potential a therapeutic target for the treatment of SLE.

Once stimulated, type I IFN binds to type I interferon receptors(IFNARs) to initiate downstream signaling via activation of the janus kinase (JAK)/STAT pathway, phosphorylation and activation of STAT-1 and STAT-2, and transcription of IFN-stimulated genes (28). There has been growing evidence to indicate that RhoA mediates phosphorylation of STAT-3 and STAT-5 in several cell types via ROCK (25), and that targeted RhoA-ROCK inhibition modulates STAT-3 phosphorylation to shift the pathologic Th17/Treg imbalance in patients with lupus (24). We found RhoA siRNA to reduce type I IFN-stimulated phosphorylation of STAT-1 but not STAT-2, leading to a downregulation of the IFN response. We confirmed RhoA inhibition to reduce expression of the type I IFN-responsive genes IFIT3, OAS1 and CXCL10, which are up-regulated in SLE patients. Conversely, the forced overexpression of RhoA upregulated ISRE and gene expression. The RhoA/ROCK inhibitor in turn reduced type I IFN-induced STAT-1 phosphorylation and ISRE reporter gene expression, as well as OAS1 and CXCL10 in human lupus PBMCs. These observations extend the observations of Gamal Badr and co-workers regarding RhoA activation by type I interferon (41), and together support a model for a positive feedback loop whereby type I IFN stimulation of RhoA contributes to the sustained and pathologic overactivation type I IFN signaling. Conceivably, interruption of this feedback pathway by the pharmacologic targeting RhoA could provide a means to reset type I IFN activation in a manner that would therapeutically beneficial in lupus but preserve necessary physiologic responses to infection.

## Conclusions

our research elucidates the positive regulatory role of RhoA in the activation of the type I IFN response pathway. we demonstrate the potential therapeutic implications of reducing RhoA expression levels or using RhoA/ROCK inhibitor Y27632 in attenuating aberrant type I IFN signaling in SLE.

## Data Availability

Data that was used in this study are available from the corresponding author wei fan upon reasonable request.

## Abbreviations

SLE: Systemic lupus erythematosus
ISRE: IFN-stimulated response element
IFN: Interferon
ISGs: IFN-stimulated genes
STAT-1: Signal transducer and activator of transcription 1
PBMCs: Peripheral blood mononuclear cells
CXCL10: C-X-C motif chemokine ligand 10
OAS1: 2’-5’-oligoadenylate synthetase 1
MX1: MX dynamin like GTPase 1
IFIT3: Interferon induced protein with tetratricopeptide repeats 3
SLEDAI: Systemic Lupus Erythematosus Disease Activity Index
JAK: Janus kinase
